# Expanding the pH range of glutamate decarboxylase from *L. pltarum* LC84 by site-directed mutagenesis

**DOI:** 10.3389/fbioe.2023.1160818

**Published:** 2023-04-13

**Authors:** Lijuan Yang, Xian Zhang, Jing Chen, Yao Zhang, Zhiping Feng

**Affiliations:** ^1^ College of Bioengineering, Sichuan University of Science and Engineering, Yinbin, China; ^2^ Liquor Making Bio-Technology and Application of Key Laboratory of Sichuan Province, Sichuan University of Science and Engineering, Yibin, China; ^3^ Faculty of Quality Management and Inspection and Quarantine, Yibin University, Yibin, China

**Keywords:** glutamate decarboxylase, molecular modification, specific enzyme activity, L-glutamate, reaction pH

## Abstract

**Introduction:** Glutamate decarboxylase is a class Ⅱ amino acid decarboxylase dependent onpyridoxal-5′-phosphate (PLP), which catalyzes the decarboxylation of substrateL-glutamate (L-Glu) to synthesize γ-aminobutyric acid (GABA). The low activity ofglutamic acid decarboxylase (GAD) and its ability to catalyze only under acidicconditions limit its application in biosynthesis of GABA.

**Methods:** Taking glutamic acid decarboxylase from *Lactobacillus plantarum*, which produces GABA, as the research object, the mutation site was determined by amino acid sequence analysis of GAD, the mutation was introduced by primers, and the mutant was constructed by whole plasmid PCR and expressed in *Escherichia coli*. Then, the enzymatic properties of the mutant were analyzed. Finally, the three-dimensional structure of the mutant was simulated to support the experimental results.

**Results and Discussion:** In this case, mutants E313S and Q347H of glutamate decarboxylase from *L. pltarum* LC84 (LpGAD) were constructed by targeted mutagenesis. Compared with the wild-type, their enzyme activity increased by 62.4% and 12.0% at the optimum pH 4.8, respectively. In the range of pH 4.0–7.0, their enzyme activity was higher than that of the wild-type, and enzyme activity of mutant E313S was 5 times that of the wild-type at pH 6.2. Visualization software PyMOL analyzed the 3D structure of the mutant predicted by homologous modeling, and the results showed that mutant E313S may broadened the reaction pH of LpGAD through the influence of surface charge, while mutant Q347H may broadened the reaction pH of LpGAD through the stacking effect of aromatic rings. In a word, mutants E313S and Q347H were improved the enzyme activity and were broadened the reaction pH of the enzyme, which made it possible for it to be applied in food industry and laid the foundation for the industrial production of GABA.

## Highlights

➢ Enhancement of GAD enzymatic activity by site-directed mutagenesis.
➢ GAD mutants can be worked in a wider ph range by site-directed mutagenesis.
➢ Homology modeling predicted the structure of GAD mutants, and visualization software PyMOL analyzed why GAD mutants could work in a wider pH range.

## 1 Introduction

γ-Aminobutyric acid (GABA), also known as aminobutyric acid, is a natural non-protein amino acid (Sze, 1979; Hua et al., 2020). GBGA has prominent physiological functions, which can promote the transmission of cerebral cortex cell signals, inhibit epilepsy, improve sleep, enhance memory and delay the aging of brain function (Cho et al., 2019; Huang et al., 2022; Lim et al., 2018; Soltani et al., 2011).

**FIGURE 1:**
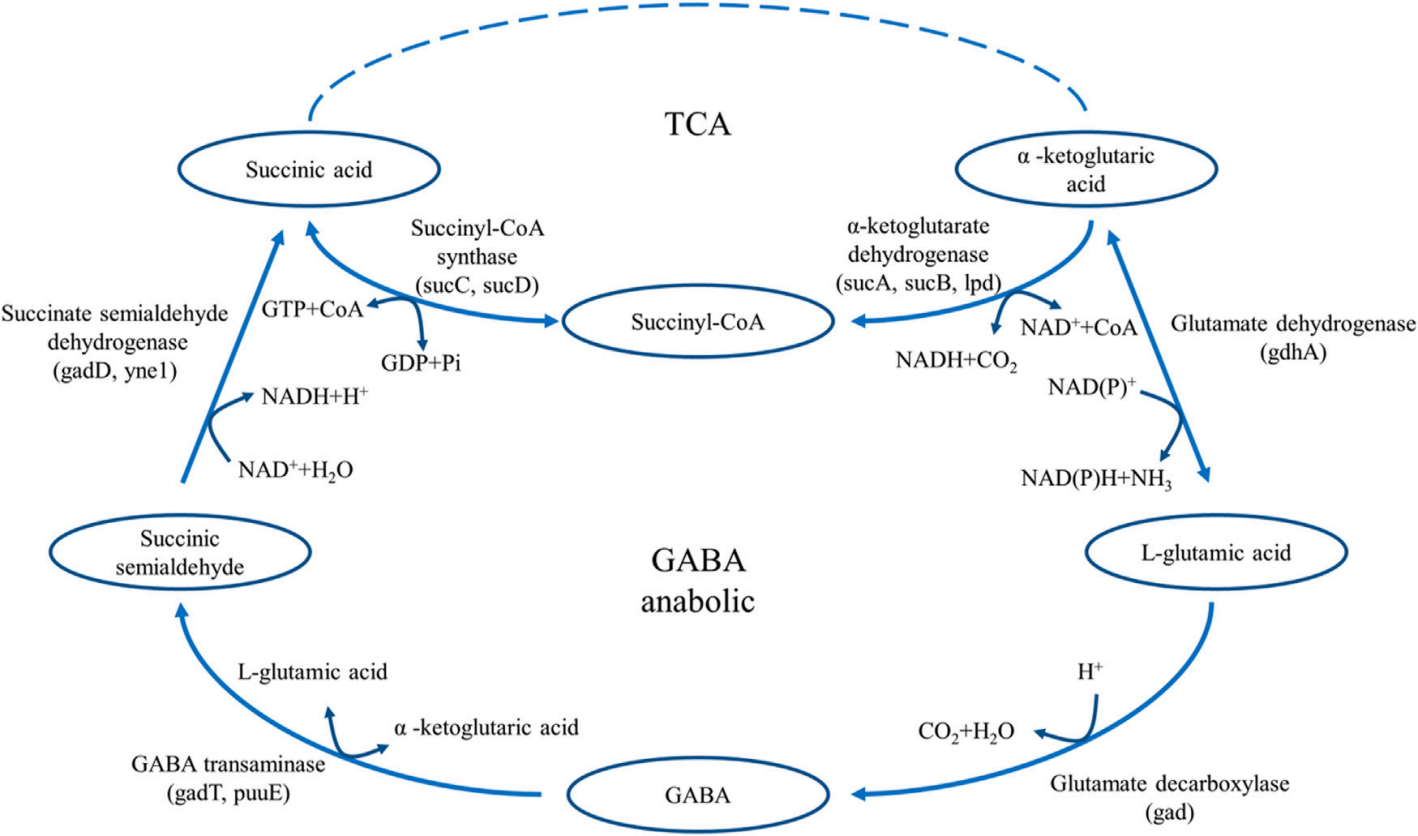
The pathway of GABA synthesis and metabolism in *E. coli*.

Glutamate decarboxylase (GAD, EC 4.1.1.15) is widely present in many animals, plants, and microorganisms (Shin et al., 2014; Sarasa et al., 2020; Sun et al., 2021). It is the only enzyme that catalyzes the production of GABA from L-glutamic acid, and plays an important role in the food and pharmaceutical industries (Wang et al., 2018). GAD is a pyridoxal 5-phosphate (PLP)-dependent enzyme that catalyzes the decarboxylation of L-glutamic acid to form GABA (Huang et al., 2018; Sandmeier et al., 1994). In microorganisms, the decarboxylation reaction of GAD is an important reaction to maintain the acid-base balance. By consuming ATP, GAD transfers a molecule of H^+^ inside the cell to the outside of the cell and converts L-glutamic acid into GABA to maintain the relative acid-base balance in the cell (Figure 1) (Feehily and Karatzas, 2013). In the catalytic reaction of GAD, the consumption of H^+^ in the reaction system leads to the increase of pH, thus inhibiting the catalytic activity of GAD. (Capitani et al., 2003). Studies have shown that L-glutamic acid is the GAD-specific substrate in the fermentation production of GABA, and the optimal pH of GAD-catalysed decarboxylation is usually between 3.8 and 4.5 (Lim et al., 2022; Son et al., 2022).

Most of the modification of GAD is to enhance its thermal stability, such as Fan et al. (2018) performed site-directed saturation mutagenesis of the N-terminal residues of GadB from *Escherichia coli* to improve its thermostability, and the triple mutant (M6, Gln5Ile/Val6Asp/Thr7Gln) showed higher thermostability. Hua et al. (2020) stabilized variants of the GAD from *Lactobacillus brevis* were constructed by consensus mutagenesis. However, little has been reported about broadening the range of GAD pH. At present, in order to solve the problem of GAD intolerance in neutral conditions, researches are mainly carried out from two aspects. First, a special buffer is used as the reaction system to maintain a relatively stable pH for a long time. Lammens et al. (2009) improved the catalytic efficiency of GAD by enzyme immobilization of calcium alginate. L-glutamic acid is used as both substrate and acid medium to regulate pH of the reaction system in order to maintain the acidic condition of GAD decarboxylation reaction. Second, GAD was mutated to broaden its reaction pH range. Thu Hoet al. (2013) obtained a Glu89Gln/His465Ala double mutant with a wider pH range by modifying GAD derived from *E. coli*. In addition, Shi et al. (2014) combined directed evolution and site-directed mutagenesis to modify GAD from *L. brevis* Lb85 and obtained three excellent mutants GadB^E312S^, GadB1^T17I/D294G/Q346H^ and GadB1^T17I/D294G/E312S/Q346H^. Their activity was 1.54–3.94 times higher than that of the wild type. Anyhow, enzymes are required to be very efficient at the right temperature and pH in the enzyme catalysis industry, too much acid or base fermentation liquid can corrode the fermenter, which is not conducive to industrial production. Therefore, the molecular modification of GAD in this study mainly focuses on two aspects, one is to improve the catalytic activity of GAD, and the other is to expand the catalytic activity and catalytic efficiency of GAD in a neutral environment.

## 2 Materials and methods

### 2.1 Strains, plasmids and mediums

The strains and plasmids used in this study are shown in Table 1, and the primers are shown in Table 2. *E. coli* strains were grown in Luria-Bertani (LB) medium supplemented with 50 mg/L kanamycin for protein expression.

**TABLE 1:** Strains and plasmids used in this study.

Strain or plasmid	Description	Source
Strains	Use for heterologous protein expression	TransGen Biotech
*E. coli* BL21(DE3)		
*E. coli* BL21(DE3)/pET-28a-*gad*	Expression strain carrying plasmid pET-28a-gad	This study
*E. coli* BL21(DE3)/pET-28a-*gad* ^D295G^	Expression strain carrying plasmid pET-28a-*gad* ^D295G^	This study
*E. coli* BL21(DE3)/pET-28a-*gad* ^S308N^	Expression strain carrying plasmid pET-28a-*gad* ^S308N^	This study
*E. coli* BL21(DE3)/pET-28a-*gad* ^E313S^	Expression strain carrying plasmid pET-28a-*gad* ^E313S^	This study
*E. coli* BL21(DE3)/pET-28a-*gad* ^Q347H^	Expression strain carrying plasmid pET-28a-*gad* ^Q347H^	This study
Plasmids		
pET-28a-*gad*	Overexpression of wild type in *E.coli*	This study
pET-28a-*gad* ^D295G^	Overexpression of mutant D295G in *E.coli*	This study
pET-28a-*gad* ^S308N^	Overexpression of mutant S308N in *E.coli*	This study
pET-28a-*gad* ^E313S^	Overexpression of mutant E313S in *E.coli*	This study
pET-28a-*gad* ^Q347H^	Overexpression of mutant Q347H in *E.coli*	This study

**TABLE 2:** Primers used in this study.

Primers	Sequence (5′-3′)
D295G-F	GgT​CGT​CAG​TTT​TTA​CCG​CCA​GAA​T
D295G-R	ACG​CCA​AAC​GAC​CCA​GCC​G
S308N-F	AaT​TAT​TTA​GGT​GGG​GAG​TTG​CCG​A
S308N-R	AAC​TTT​GAA​GAC​TAA​TTC​TGG​CGG​T
E313S-F	tcG​TTG​CCG​ACA​ATG​GCG​ATC
E313S-R	CCC​ACC​TAA​ATA​ACT​AAC​TTT​GAA​GAC​TAA​TTC​TGG​C
Q347H-F	CAc​ACA​AAG​ACT​CAC​GAT​GTT​GCC​C
Q347H -R	AAT​CTC​GCG​GTA​ACC​GTC​CAT​AC

Note: lowercase letters indicate mutant base.

### 2.2 Homology modeling of GAD and its variants

Multiple sequence alignment of the amino acid sequence of LpGAD from *L. plantarum* LC84 was performed using the Clustal Omega program (http://www.ebi.ac.uk/Tools/msa/clustalo/) (Madeira et al., 2022). The LpGAD alignment results were beautified using the ESPript 3.0 web server (http://espript.ibcp.fr/ESPript/cgi-bin/ESPript.cgi) (Robert and Gouet, 2014). Three-dimensional structural models of LpGAD wild-type and its variants were generated using SWISS-MODEL (https://swissmodel.expasy.org/) (Waterhouse et al., 2018). Molecular modeling was visualized and analyzed using PyMOL 2.1 (https://pymol.org/2/).

### 2.3 Construction of mutant plasmids and strains

Using the recombinant plasmid pET-28a-gad as the template, the concentration was adjusted to 50 ng/μL, and the four pairs of mutation primers in Table 2 were used for PCR amplification, and then used KOD-Plus-Mutagenesis kit (TOYOBO) to obtain mutant plasmids pET-28a-*gad* ^D295G^, pET-28a-*gad* ^S308N^, pET-28a-*gad* ^E313S^ and pET-28a-*gad* ^Q347H^ containing each mutation site. Specifically, DpnI enzyme was added to the PCR product to remove the template plasmid pET-28a-*gad*, and then the product was formed into a circular mutant plasmid under the action of T4 Polynucleotide Kinase and Ligation high.

The recombinant plasmid was transformed into *E. coli* BL21 (DE3) to construct recombinant *E. coli* strains BL21 (DE3)/pET-28a-*gad*, BL21 (DE3)/pET-28a-*gad* ^D295G^, BL21 (DE3)/pET-28a-*gad* ^S308N^, BL21 (DE3)/pET-28a-*gad* ^E313S^ and BL21(DE3)/pET-28a-*gad* ^Q347H^.

### 2.4 Expression of recombinants in *E. coli*

Recombinant *E. coli* strains BL21 (DE3)/pET-28a-*gad*, BL21(DE3)/pET-28a-*gad* ^D295G^, BL21(DE3)/pET-28a-*gad* ^S308N^, BL21(DE3)/pET-28a-*gad* ^E313S^ and BL21 (DE3)/pET-28a-*gad* ^Q347H^ were inoculated in LB medium, 50 mg/L kanamycin was added, and the strains were cultured with shaking at 37°C for 12 h at 180 rpm. Then, the culture was inoculated into new LB medium at 2% inoculum and incubated at 37°C for 2.5 h, and then isopropyl thiogalactoside (IPTG) was added at a final concentration of 0.5 mmol/L. Incubate for 8 h at 16°C and 160 rpm for protein expression. *E. coli* strains were harvested by centrifugation (4,000 × g, 8 min), washed 3 times with PBS buffer (pH 7.4), and resuspended in the same buffer. Then at 120 W, the *E. coli* strains suspension was disrupted by sonication for 10 min at 5 s intervals for 5 s.

### 2.5 Purification of LpGAD and its variants

Recombinant LpGAD and its variants were purified using nickel column affinity chromatography. The nickel column was equilibrated with nickel column equilibration buffer (250 mmol/L NaCl, 5 mmol/L imidazole in 20 mmol/L, pH 7.4 PBS buffer) before purification. Then use elution buffer (containing 10–250 mmol/L imidazole) to elute and purify the target protein. The eluted protein was dialyzed against dialysis buffer (pH 4.6). The eluate was detected by sodium dodecyl sulfate-polyacrylamide gel electrophoresis (SDS-PAGE). Protein concentration was determined using the BCA method.

### 2.6 Determination of enzyme activity

The enzyme activity of LpGAD was determined by the Berthelot colorimetric method (Kitaoka and Nakano, 1969). The principle is that in the presence of phenol, sodium hypochlorite and free ammonia, a highly sensitive color reaction will occur. The trace ammonia and salts in the system can be determined according to the color changes of reaction. The content of similar substances, this method is used for the detection of GABA, its accuracy is compared with HPLC, the error is about 4.9% (Xiao-Mei et al., 2006). Take 0.6 mL of the mixed standard solution, add 0.1 mL of 1 mol/L Na_2_CO_3_ solution, 0.5 mL of 0.2 mol/L pH 10.0 borate buffer, and 1 mL of 6% phenol, and mix well. After that, 1 mL of NaClO solution was added, mixed evenly, left for 4 min, immediately boiled in water for 10 min, and ice-bathed for 20 min. After the solution turned blue-green, 2 mL of 60% ethanol was added, mixed and placed in a water bath at 20°C for 40 min. Using 10 mmol/L L-Glu as blank control, the OD value was measured at a wavelength of 640 nm. Take GABA content as abscissa and OD_640_ absorbance as ordinate to make standard curve. The standard curve equation is y = 0.0784x − 0.008 with R^2^ value of 0.9991.

Pipette 200 μL of LpGAD enzyme solution and PBS buffer (blank) into a colorimetric tube, and add 400 μL of substrate reaction solution Macilvaine buffer (pH 4.8, with 0.02 mol/L L-Glu and 0.4 mmol/L pyridoxal phosphate). After mixing, the reaction was carried out at 45°C for 30 min. After the reaction, it was taken out and placed in an ice bath to terminate the reaction. The GABA produced by the reaction was detected by Berthelot method. One unit of enzyme activity (U) is defined as the amount of enzyme that can convert 1 μmol of substrate in 1 min at 45°C in the reaction solution.

### 2.7 Characterization of LpGAD and its variants

The substrate buffer solutions (0.2 mol/L Na2HPO4 − 0.1 mol/L citric acid, pH 3.0–7.0) are prepared to determine the optimum pH. Mix the enzyme solution with a certain amount of substrate buffer solution preheated at 45°C, and react at 45°C for 30 min then detect the content of GABA.

For the determination of pH stability, the enzyme solution was mixed with a certain amount of substrate buffer solution and then incubated at 4°C for 24 h. After incubation, the residual activity was measured. The stability of LpGAD under different pH conditions was investigated.

## 3 Results

### 3.1 Selection of mutated amino acid residues

**FIGURE 2:**
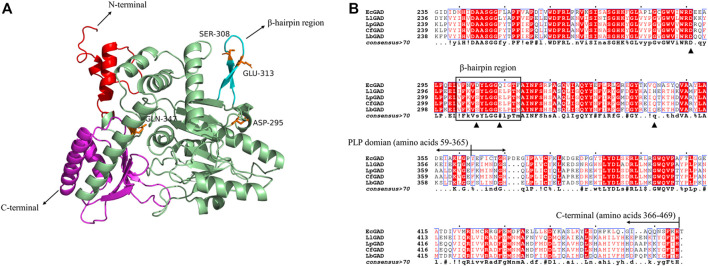
3D structural modeling and amino acid sequence analysis of LpGAD. **(A)** LpGAD structure predicted using the SWISS-MODEL server, using the GAD of *Lactobacillus brevis* (PDB ID 5GP4) as a template. Red: amino acids 1–58 at the N-terminal; palegreen: PLP domain; cyan: β-hairpin region (amino acids 304–317); magenta: amino acids 366–469 at the C-terminal; orange: mutated amino acid site. **(B)** LpGAD amino acid sequence analysis. EcGAD, LlGAD, LpGAD, CfGAD, and LbGAD represent the GAD of *Escherichia coli*, *Lactococcus lactis*, *Lactobacillus* plantarum LC84, Companilactobacillus futsaii and *Lactobacillus brevis* CGMCC 1306, respectively; triangles represent mutated amino acid sites; black boxes represent β-hairpin region (amino acids 304–317).

In bacterial, the subunit structure of GAD consists of three domains, namely, N-terminal, PLP binding domain and C-terminal. In this study, we aimed to improve the catalytic activity and broaden the pH range of *L. plantarum* LC84 GAD. The LpGAD of *L. plantarum* LC84 was modeled using SWISS-MODEL. The template used *L. brevis* CGMCC 1306 GAD crystal structure (PDB code: 5GP4) (Huang et al., 2018). The template shared 82.69% amino acid sequence identity with LpGAD (Figure 2A).

In 2003, Capitani et al. (2003) analyzed the GAD structure of *E. coli* and found that the GAD 3D structure was basically consistent in neutral and acidic pH, and only changed in the N-terminal, C-terminal and β-hairpin structure (amino acid residues 300–313). De Biase and Pennacchietti (2012) analyzed and summarized the GAD of *E. coli*, and found that the 15 amino acid residues in the N-terminal, the last 15 residues in the C-terminal (347–466) and the β-hairpin structure (300–313) were found to be directly related to acid-base adaptability, enzyme activity and catalytic potency of GAD. This provides a reference for us to modify acid-base adaptability of *Lactobacillus* plantarum LC84 GAD catalysis. At present, there have been successful cases of broadening the pH range of GAD catalysis by molecular modification. Yu et al. (2012) obtained mutant S307N from *L. brevis* GAD, and its catalytic efficiency at pH 6.0 was twice that of the wild enzyme. Shi et al. (2014) modified the GAD of *L. brevis* Lb85 and obtained mutants D294G, E312S and Q346H with improved specific enzyme activity at pH 6.

Therefore, amino acid residues of LpGAD (amino acids at positions 295, 308, 313 and 347) corresponding to the *L. Brevis* GAD at positions 294, 308, 312 and 346 were selected for mutation (Figure 2B). The variants D295G, S308N, E313S and Q347H were obtained, in which S308N and E313S were located in β-hairpin structure (amino acid residues 304–317) of LpGAD.

### 3.2 Construction of variants and analysis of enzyme activity

**FIGURE 3:**
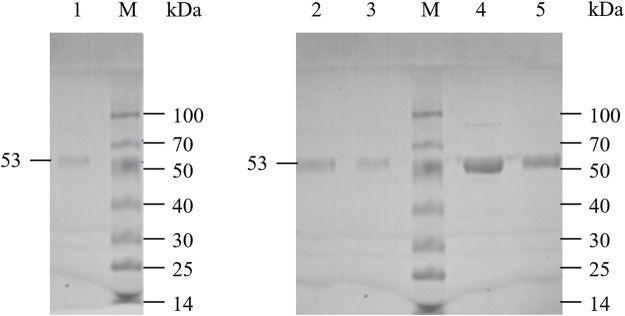
SDS-PAGE analysis of LpGAD and its variants expression. Lane M, protein molecular weight markers; lane 1, purified LpGAD; lane 2, purified mutant D295G; lane 3, purified mutant S308N; lane 4, purified mutant E313S; lane 5, purified mutant Q347H.

LpGAD and its variants D295G, S308N, E313S and Q347H were purified using a nickel affinity chromatography column. After purification, the molecular weight of LpGAD and its variants was determined to be 53 kDa by SDS-PAGE (Figure 3). The enzyme activity activities of variants D295G, S308N, E313S and Q347H to L-Glu at pH 4.8 were checked (Table 3). Compared with wild-type LpGAD, the specific activities of the variants D295G and S308N decreased by 26.2% and 33.8%, respectively. However, the specific activities of the variants E313S and Q347H were increased by 62.4% and 12.0%, respectively.

**TABLE 3:** Determination of specific activity of LpGAD and its variants.

Variants	Specific activity (U/mg)
WT	41.660 ± 0.64
D295G	20.745 ± 0.72
S308N	25.578 ± 0.25
E313S	67.650 ± 0.58
Q347H	46.659 ± 0.66

### 3.3 Analysis of optimal pH and pH stability of LpGAD variants

**FIGURE 4:**
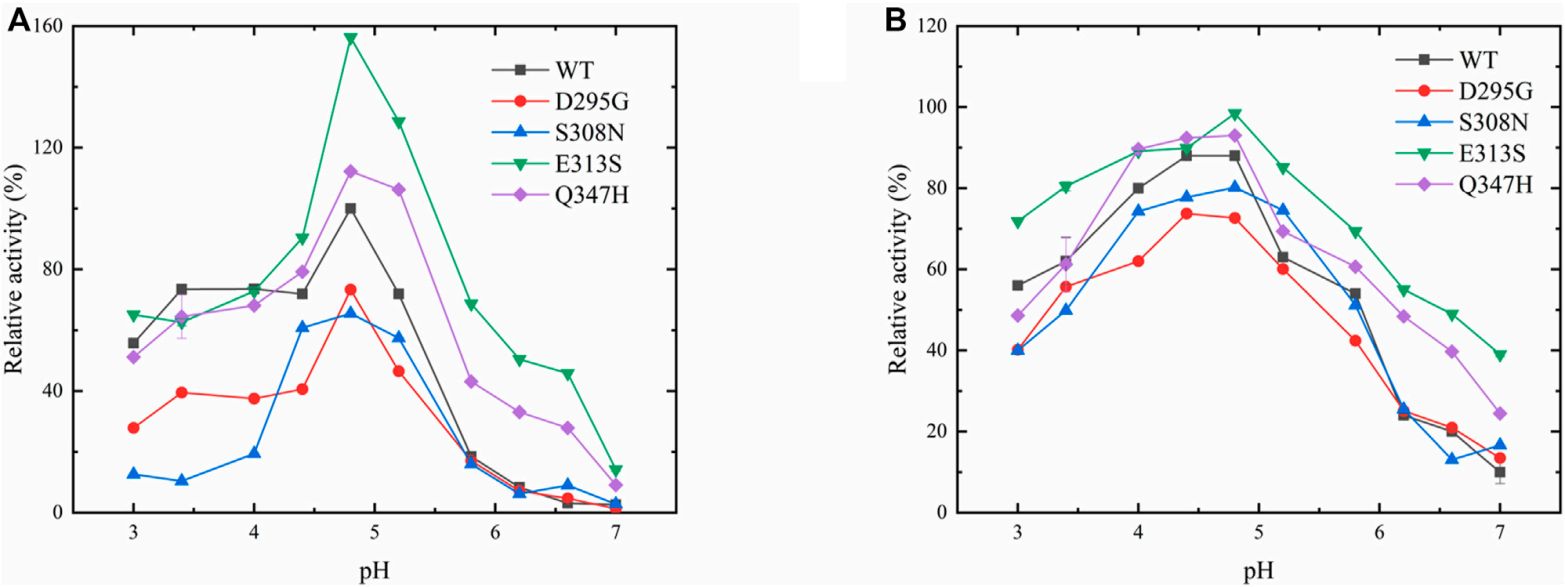
Effects of pH on the enzymatic activity of LpGAD and its variants. **(A)** Effect of pH on enzyme activity; **(B)** Effect of pH on enzyme stability.

The enzyme activity and stability of the variants were measured at different pH (Figure 4). The enzyme activity of wild-type LpGAD at pH 4.8 was 100%, and the enzyme activities of LpGAD and its mutants under various pH conditions were calculated, as shown in Figure 4A. At pH 4.8, the enzyme activity of the mutant D295Gcould reach 70% of that of the wild type. During pH 5.8–7.0, the enzyme activity of the mutant D295G was basically consistent with that of the wild type. When pH was 4.8, the enzyme activity of the mutant S308N was the lowest, only 60% of that of the wild type. The enzyme activity of the mutant S308N under neutral conditions had no obvious change compared with the wild type. The mutants D295G and S308N did not enhance the pH adaptability of LpGAD, which was contrary to the results of the mutants D294G and S308N from *L. brevis* GAD (Yu et al., 2012; Shi et al., 2014). The possible reason is that *L. brevis* GAD and *L. plantarum* LC84 GAD differ in some amino acid residues near the enzyme active center, which may lead to different mutation results. For mutant E313S, in the range of pH 3.0–4.0, its enzyme activity is basically consistent with that of the wild type, in the range of pH 4.0–4.8, its enzyme activity is higher than that of the wild type, and reaches the highest at pH 4.8, which is about 1.6 times of that of the wild type. In the range of pH 4.8–7.0. The enzyme activity of mutant E313S showed a downward trend, but the enzyme activity was higher than that of the wild type. At pH 6.2, the enzyme activity of mutant E312S increased about 5 times compared with that of the wild type. In the range of pH 3.0–4.4, the enzyme activity of Q347H mutant was not different from that of the wild type, but kept the same trend with that of the wild type. The enzyme activity of Q347H mutant reached the highest at pH 4.8, which was higher than that of the wild type. The enzyme activity of Q347H mutant increased about 2 times compared with that of the wild type at pH 6.2. The enzyme activities of mutants E313S and Q347H were higher than those of the wild type in the range of pH 3.0–7.0. Thus, the E313S and Q347H mutants successfully broadened the pH of the GAD catalyzed reaction.

To test the acid stability of LpGAD mutants, the four mutants were stored at 4°C, pH 3.0–7.0, for 24 h. The initial enzyme activity of each mutant was 100%, and the changes of enzyme activity were detected. As can be seen from Figure 4B, the wild-type and its mutants have similar pH stability, with the strongest stability at the optimum pH 4.8. The mutants E313S and Q347H maintain more than 80% enzyme activity, while mutants D295G, S308N and wild-type maintain about 70% enzyme activity. In the range of pH 4.8–5.8, mutant E313S maintained about 60% enzyme activity, mutant Q347H maintained more than 40% enzyme activity, wild-type LpGAD, D295G and S308N were significantly inactivated, and the enzyme activity was low. Therefore, compared with the wild-type, the E313S and Q347H mutants maintained their acidic stability better under weak acidic or neutral conditions.

### 3.4 Structural simulation analysis of LpGAD mutants

**FIGURE 5:**
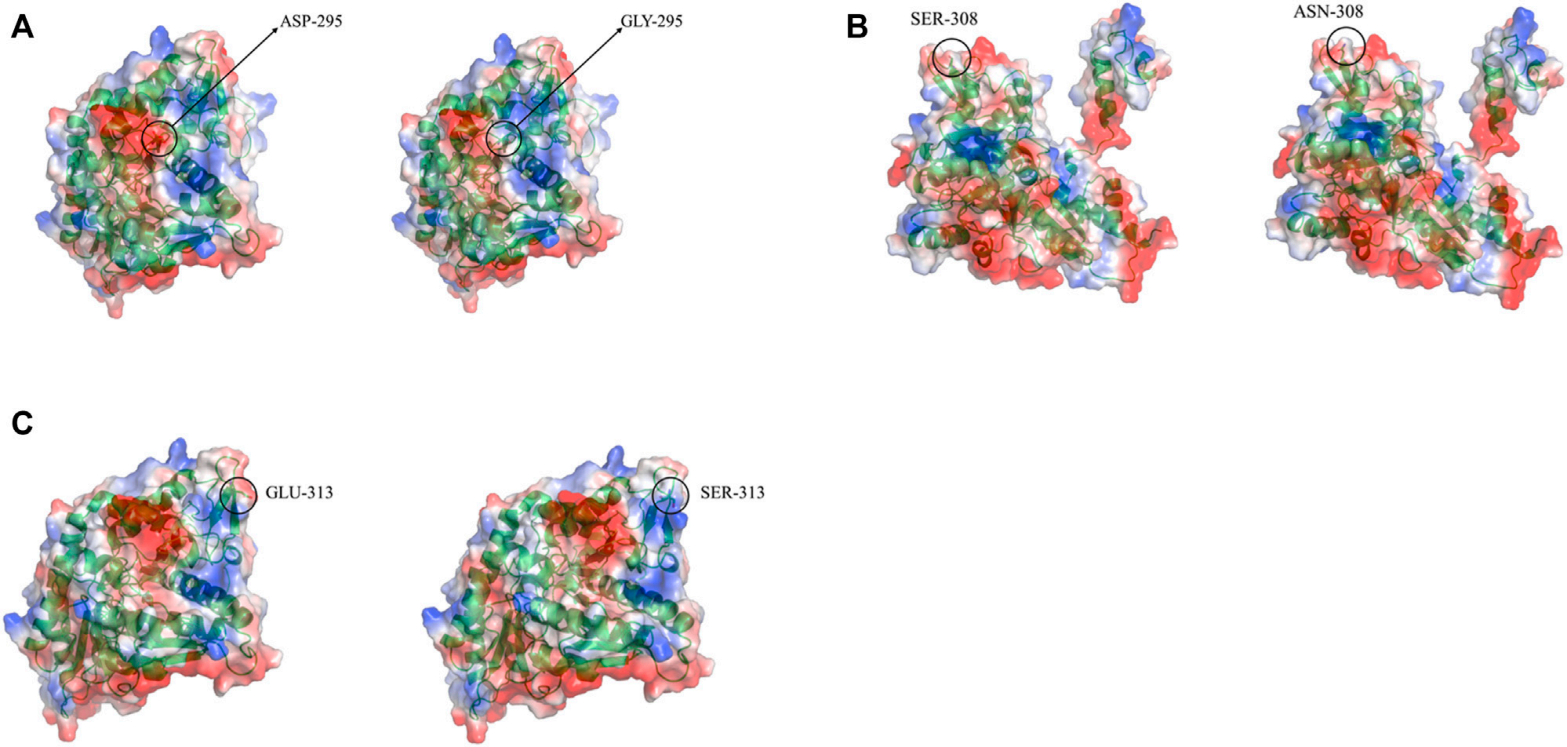
Changes in electrostatic potential of mutant sites before and after LpGAD mutation. **(A)** The change of the electrostatic potential of the amino acid residue at position 295 before and after mutation; **(B)** The change of the electrostatic potential of the amino acid residue at position 308 before and after the mutation; **(C)** The change of the electrostatic potential of the amino acid residue at position 313 before and after the mutation. Left is wild type, right is LpGAD mutant, blue represents positive potential, white represents neutral potential, red represents negative potential.

**FIGURE 6:**
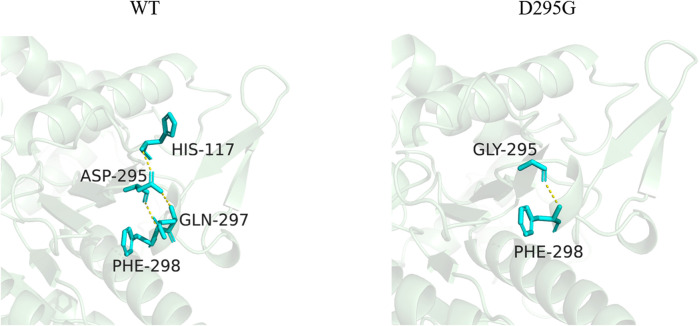
Changes of hydrogen bonds between amino acid residues at position 295 and surrounding amino acids before and after mutation. The yellow dotted lines in the figure represent hydrogen bonds.

Three-dimensional models of LpGAD mutants were constructed using SWISS-MODEL and analyzed using PyMOL software. By changing the surface charge of the protein by site-directed mutagenesis, the catalytic efficiency of the enzyme can be improved and the optimum pH can be changed (Russell and Fersht, 1987). The mutation sites of mutants D295G, S308N and E313S are all located on the surface of LpGAD protein, in which the change of amino acid residues of mutants D295G and E313S changes the surface charge of protein and enhances the electropositivity of protein surface (Figures 5A, C). The enhancement of positivity is conducive to the stability of the protein under neutral and weakly acidic conditions (Neves-Petersen et al., 2001). But the mutation of site 295 from Asp to Gly reduces the number of hydrogen bonds from 3 to 1. Hydrogen bonding is an important force for stabilizing the secondary structure of enzyme proteins (Li et al., 2005). The number of hydrogen bonds in the mutant D295G was reduced, which may be the reason for the decreased enzyme activity and stability (Figure 6). The mutant S308N mutated Ser to Asn, failed to change the surface charge of this site, and the enzyme activity and stability were not improved (Figure 5B).

**FIGURE 7:**
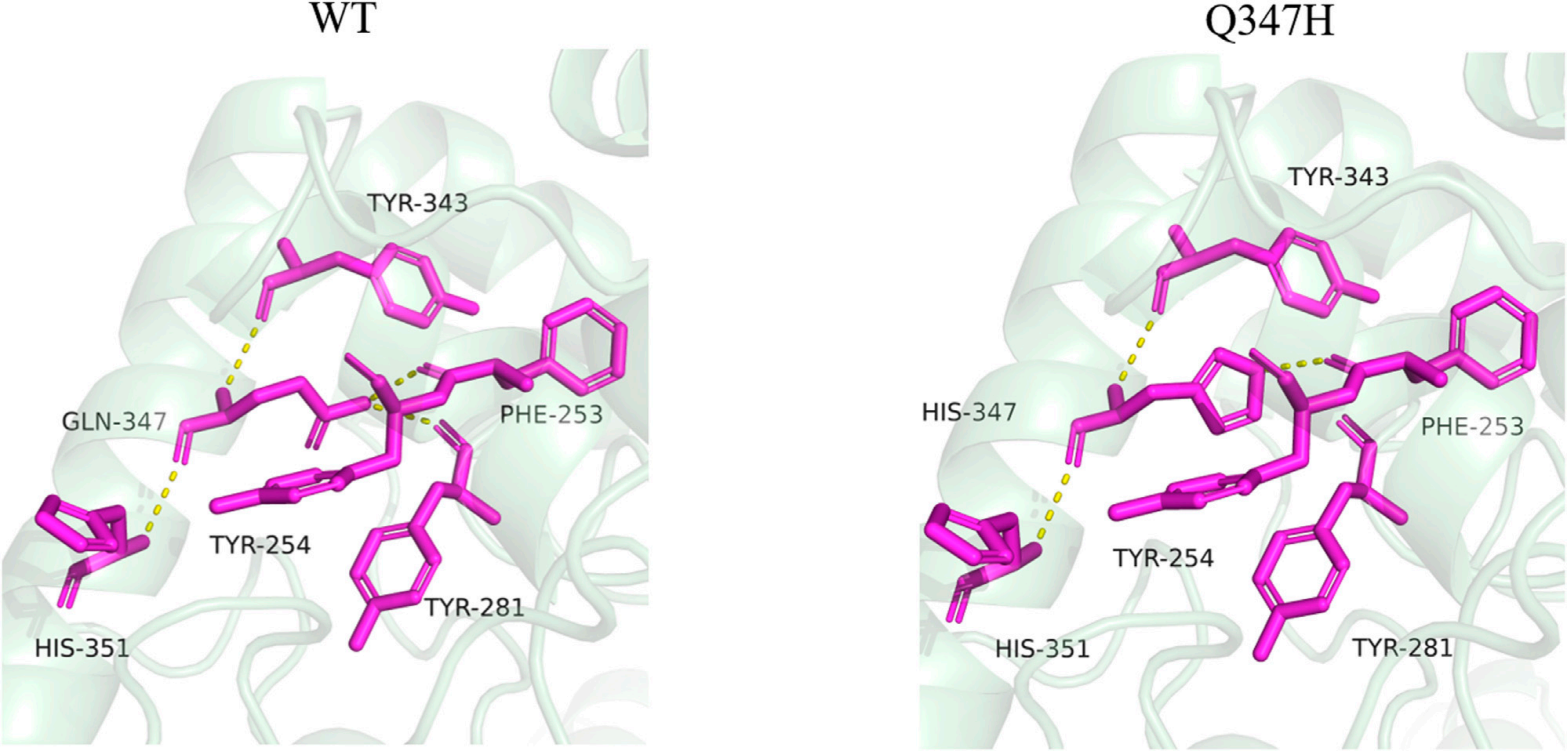
Changes of hydrogen bonds between amino acid residues at position 347 and surrounding amino acids before and after mutation. The yellow dotted lines in the figure represent hydrogen bonds.

By analyzing the interaction between the mutant site and the surrounding amino acids before and after mutation, it was found that mutant Q347H relieved the hydrogen bond between Gln347 and Tyr281, and mutated Gln into His, introducing the aromatic ring (heterocycle). As can be seen from Figure 7, most of the amino acids having an aromatic ring in the vicinity of position 347 are found. It has been reported in the literature that the accumulation of aromatic rings can regulate the enzyme activity decrease at neutral pH and increase at alkaline pH (Qin et al., 2008), which is consistent with the enzymatic properties of our mutant Q347H.

## 4 Discussion

Glutamate decarboxylase (GAD) is the only enzyme capable of converting glutamate (L-Glu) into γ-aminobutyric acid. Gamma-aminobutyric acid (GABA) is a non-protein amino acid that exists widely in living organisms and has many important physiological functions, such as lowering blood pressure, sedation and preventing senile dementia. Therefore, GABA has huge application potential in pharmaceutical, food, healthcare and other industries. At present, the capacity of domestic GABA-producing wild strains is low. At the same time, in the process of fermentation production, the optimum pH of GAD is low, generally between 4.0 and 5.0. When the pH reaches 6.0, the enzyme will be deactivated due to depolymerization, which is not conducive to industrial application.

In this study, in order to broaden the pH adaptability of GAD derived from *L. Pantarum* LC84, the molecular modification of wild-type LpGAD was carried out, and four mutants D295G, S308N, E313S and Q347H were successfully constructed. The study of enzymatic properties showed that the enzyme activity of mutant E313S was about 1.6 times that of the wild type at pH 4.8, and the activity of mutant E313S was about 5 times higher than that of the wild type at pH 6.2, and the enzyme activity of mutant E313S was still more than 60% in the range of 4.8–5.8. At pH 4.8, the enzyme activity of mutant Q347H was slightly higher than that of wild type. At pH 6.2, the enzyme activity of mutant Q347H increased by about 2 times. In the range of pH 4.8 to 5.8, the enzyme activity of mutant Q347H maintained more than 40%.

In conclusion, we successfully constructed LpGAD mutants E313S and Q347H, whose enzyme activity was enhanced compared with the wild-type, and the reaction pH was successfully broadened. Mutants E313S and Q347H have great potential for GABA production.

## Data Availability

The original contributions presented in the study are included in the article/Supplementary Material, further inquiries can be directed to the corresponding author.
